# Inhibition of *Neisseria gonorrhoeae* complement-mediated killing during acute gonorrhea is dependent upon the IgG2:IgG3 antibody ratio

**DOI:** 10.1128/mbio.03367-23

**Published:** 2026-01-30

**Authors:** Samantha A. McKeand, Sian E. Faustini, Alex Cook, Nikki Kennett, Mark T. Drayson, Adam F. Cunningham, Ian R. Henderson, Christoph M. Tang, Jonathan D. C. Ross, Jeffrey A. Cole, Amanda E. Rossiter-Pearson

**Affiliations:** 1 Institute of Microbiology and Infection, University of Birmingham 1724, Birmingham, United Kingdom; 2 Sir William Dunn School of Pathology, University of Oxford 6396, Oxford, United Kingdom; 3 Institute of Immunology and Immunotherapy, College of Medicine and Health, University of Birmingham 1724, Birmingham, United Kingdom; 4 The Binding Site, Birmingham, United Kingdom; 5 Institute for Molecular Bioscience, University of Queensland 85088, St. Lucia, Queensland, Australia; 6 Sexual Health and HIV, University Hospitals Birmingham 255251, Birmingham, United Kingdom; 7 Department of Microbes, Infection and Microbiomes, School of Infection, Inflammation and Immunology, College of Medicine and Health, University of Birmingham 1724, Birmingham, United Kingdom; Georgia Institute of Technology, Atlanta, Georgia, USA

**Keywords:** *Neisseria gonorrhoeae*, blocking antibody, complement-mediated killing, serum resistance

## Abstract

Excessive binding of antibodies to the bacterial cell surface can paradoxically increase resistance of some Gram-negative pathogens to complement-mediated killing (CMK). We examined the CMK of 336 *Neisseria gonorrhoeae* isolates from 283 participants recruited to a clinical trial. Serum bactericidal assays revealed 3% (9/283) of the autologous participant sera blocked CMK. Gonococci isolated from these participants were resistant to the autologous host serum, but sensitive to pooled healthy control sera (HCS) and protected by autologous host serum in a 1:1 mixture with HCS. Analysis of clinical metadata showed that there was a significantly higher proportion of blocking sera found in participants with urethral infection and from men within the transmission network of men who have sex with women, when compared to the whole cohort. Following antibody purification from participants with blocking sera (5/9), total IgG protected autologous isolates from HCS-mediated killing. A closer examination of IgG subclasses using whole gonococcal cell ELISAs revealed a significant correlation between increased IgG2 binding and decreased IgG3 binding to the cell surface of isolates that were resistant to CMK. This indicates that IgG2 prevents bactericidal IgG3 from initiating CMK, with an increased IgG2:IgG3 ratio blocking CMK of gonococci. We therefore reveal a previously unrecognized mechanism by which blocking antibodies prevent CMK of *N. gonorrhoeae*.

**IMPORTANCE:**

The antigenic variation of *Neisseria gonorrhoeae* and a limited mechanistic understanding of immune responses to this bacterium have presented multiple challenges to generating a protective vaccine. Here, we use a collection of *N. gonorrhoeae* clinical isolates (*n* = 336) for a robust analysis of the host immune response to infection. We reveal a mechanism for serum resistance in which some isolates of *N. gonorrhoeae* drive the production of inhibitory IgG2 antibodies, which block the activity of IgG3 bactericidal antibodies. Importantly, an increased ratio of IgG2:IgG3 bound to the bacterium promotes serum resistance. Recently, there has been increased interest in developing a vaccine against *N. gonorrhoeae* given the observation that the licensed outer membrane vesicle-based vaccine against *Neisseria meningitidis* (MeNZB) generated some cross-protection against *N. gonorrhoeae*. Thus, the mechanism described here should guide the development of a vaccine that simultaneously prevents serum resistance and promotes serum killing of the gonococcus.

## INTRODUCTION

Gonorrhea is a sexually transmitted infection (STI) caused by the Gram-negative bacterium *Neisseria gonorrhoeae*. It is the second most common bacterial STI globally, with 82.4 million diagnosed infections reported in 2020 alone (1). Gonococcal disease typically affects the genitourinary tract of women and men, but can also be isolated from mucosal surfaces of the oropharynx and the rectum. Symptoms are more common in men than in women, with disease complications including epididymo-orchitis in men and pelvic inflammatory disease, ectopic pregnancy, and infertility in women (2, 3). Disseminated gonococcal infection (DGI) is a severe systemic disease that can occur in 0.5%–3% of individuals (4).

The emergence of antimicrobial resistance in *N. gonorrhoeae* raises the concern that untreatable gonorrhea might be inevitable (5). Therefore, there is an urgent need to develop an effective vaccine against this important human pathogen. Despite efforts spanning over a century, there is currently no FDA-approved vaccine available for gonorrhea (6), with efforts hampered by the lack of protective immunity following infection, and an incomplete understanding of how the bacterium subverts immune responses.

More recently, retrospective analysis of data from a mass vaccination campaign in New Zealand with an outer membrane vesicle (OMV) vaccine (MeNZB) against serogroup B *Neisseria meningitidis* revealed an association between immunization and an ~30% reduction in gonorrhea among young adults (7). These observations indicate that protection can be induced against *N. gonorrhoeae* and have motivated efforts to produce a vaccine specifically for *N. gonorrhoeae*.

Antibodies typically protect hosts against pathogens. However, antibody-dependent enhancement (ADE) can occur in which antibodies are directly linked with disease exacerbation (8). “Blocking” or “cloaking” antibodies can mediate ADE and have been identified in the serum of patients with Gram-negative bacterial infection, including *Salmonella enterica* serovar Typhimurium, *Pseudomonas aeruginosa*, and *Escherichia coli* (9–13). The proposed mechanisms for this phenomenon involve antibodies promoting the deposition of complement at a site distant from the bacterial surface and/or blocking the access of bactericidal antibodies to the bacterial cell surface (8). Moreover, symptoms of patients with *P. aeruginosa* infection and severe bronchiectasis improved when blocking antibodies were removed (14). In addition, earlier studies have characterized blocking antibodies against *N. gonorrhoeae* that were isolated from sera from healthy individuals (15). These antibodies blocked complement-mediated killing (CMK) of *N. gonorrhoeae* strains from patients with DGI (15, 16). Removing these blocking antibodies *in vitro* restored CMK (17, 18). Further characterization revealed that these antibodies were directed against the gonococcal outer membrane protein III, also known as RmpM (reduction modifiable protein) (18). However, the nature and indeed the prevalence of these blocking antibodies among patients with acute gonorrhea have not been well defined. Thus, little is known about whether natural infection elicits blocking antibodies in patients, and, if so, their prevalence and implications for vaccine design against *N. gonorrhoeae*.

In this study, we utilized *N. gonorrhoeae* clinical isolates (*n* = 336) and autologous serum from 283 participants collected as part of the G-ToG clinical trial (19). Using these samples, we determined whether blocking antibodies are produced by infected individuals and whether they were associated with any of the clinical features, such as gender, HIV status, sexual networks, anatomical site of infection, and recurrent infections. We found that 3% of participants harbored serum that was able to protect their autologous isolates from CMK. IgG purified from these sera was able to protect isolates from CMK mediated by healthy control sera (HCS). However, protection was variable depending on the gonococci strain; we found that this variability was associated with an increased IgG2 and decreased IgG3 binding to the bacterial cell surface. Ultimately, our results demonstrate that the mechanism underlying protection of *N. gonorrhoeae* from CMK is distinct from that of blocking antibodies against other Gram-negative bacteria, which depend on elevated titers of IgG2 alone. Instead, we show that the IgG2:IgG3 ratio binding to the bacterial cell surface determines survival of gonococci in serum.

## MATERIALS AND METHODS

### Serum and bacterial isolates

From the 720 participants recruited to the G-ToG trial (19), 336 *N*. *gonorrhoeae* isolates were collected from 283 individuals. Blood samples were obtained from participants and stored as serum by the Clinical Immunology Services (The University of Birmingham, UK). Bacterial isolates are labeled “NGxxx,” and serum isolated from the participant with the indicated isolate is referred to as “NGxxx-serum.” Upon trial entry, clinical and demographic data were collected from participants and recorded in a database developed by the Nottingham Clinical Trial Unit within the G-ToG team. Blood was taken from eight healthy human volunteers with no previous history of gonococcal infection, and equal volumes from individuals were pooled to generate HCS, which was stored at −80°C.

### Growth conditions

Bacteria were routinely grown on gonococcal base medium (GCB) agar plates containing 1% Vitox supplement (Oxoid) in a candle extinction jar for 20 h at 37°C, and single colonies were passaged onto fresh plates and grown for another 16–18 h.

### Serum bactericidal assay

Serum bactericidal assays (SBAs) were used to determine the susceptibility of gonococcal isolates to CMK. Bacteria grown overnight on GCB agar were resuspended in phosphate-buffered saline (PBS) at a final concentration of 1.5 × 10^7^ colony-forming units (CFUs) per mL; suspensions were incubated at 37°C with the autologous serum, HCS, or a 1:1 mixture of both sera. When an exogenous complement source was required, bacteria were added to a mixture of 5% baby rabbit complement (BRC) (AbD Serotec) and heat-inactivated (HI) participant serum. As a negative control, bacteria were added to PBS only and incubated under the same conditions. Aliquots were taken before incubation and at hourly intervals for 3 h, serially diluted and plated in triplicate onto GCB agar. After overnight growth at 37°C, the number of CFUs at time points was counted and relative survival calculated by comparison with colony counts at 0 h. For SBAs with purified antibody, bacteria were prepared in the same way and added to a mixture of HCS and total IgG purified from individual participant sera at the indicated concentrations in PBS.

### Construction of *N. gonorrhoeae rmpM* mutants

DNA sequences of ~1,000 base pairs (bp) up and downstream of *rmpM* were amplified using Herculase II polymerase (Agilent) from genomic DNA of target *N. gonorrhoeae* isolates using primer pairs *rmpM*_upstream_F and R, and *rmpM*_downstream_F and R (Table S1), which included ~20 bp overhang sequences complementary to an *aph(3)* cassette (20), conferring resistance to kanamycin. This cassette was amplified by PCR using primers *aph(3)*_F and *aph(3)*_R, also including ~20 bp sequences complementary to regions upstream and downstream of *rmpM*. The three PCR products were then fused by overlap PCR using PrimeSTAR GXL polymerase (Takara Bio). Amplicons were gel purified using Wizard SV Gel and PCR Clean-Up System (Promega) and used to transform target *N. gonorrhoeae* isolates. Mutants were generated by spotting 1 μg of DNA fragments onto GCB plates. Once dry, fresh isolated colonies from corresponding *N. gonorrhoeae* isolates were swabbed over the DNA spot and incubated with 5% CO_2_ at 37°C for 8 h. Growth over the spot was then swabbed onto GCB containing 80 μg/mL kanamycin and incubated with 5% CO_2_ at 37°C for 24–48 h. Deletion of *rmpM* by homologous recombination replacement with *aph(3*) was confirmed by PCR (using primers listed in Table S1) and Sanger sequencing. Mutants were further confirmed by Western blot. In brief, *N. gonorrhoeae* whole-cell lysates (10 μL) were separated on a 12% SDS-polyacrylamide gel. Proteins were transferred to Amersham Protran 0.45 μm nitrocellulose membrane (GE Healthcare) at 25 V for 30 min using the Trans-Blot Turbo system (Bio-Rad). Membranes were incubated overnight in blocking buffer (PBS with 0.5% Tween-20 and 5% skim milk) at 4°C. Primary anti-RmpM antibodies (21) were diluted 1:10,000 in blocking buffer and incubated at room temperature for 2 h. After three 10 min washes in PBS-T (PBS with 0.5% Tween-20), membranes were incubated with secondary polyclonal goat anti-mouse antibodies conjugated to horseradish peroxide (HRP) (Dako), diluted 1:10,000. After final washes, membranes were developed using Amersham ECL Western Blotting Analysis System (GE Healthcare) and exposed to Amersham Hyperfilm ECL (GE Healthcare).

### Antibody purification

Total IgG was purified from 500 µL of participant serum, flowed through a Protein G column (Cytiva) at 1 mL/min, and purified total IgG was eluted at 2 mL/min in a fraction size of 5 mL.

### Enzyme-linked immunosorbent assay

An enzyme-linked immunosorbent assay (ELISA) was used to determine IgG subclasses binding to gonococcal isolates. Bacteria grown on plates were resuspended in PBS and adjusted to an OD_600 nm_ of 0.5 to coat high-binding ELISA plates (Nunc-Immuno, ThermoScientific). After incubation overnight at 4°C, plates were blocked with 1% bovine serum albumin (BSA) in PBS for 1 h. Participant sera pre-diluted 1 in 20 were added to plates and serially diluted threefold in blocking buffer (0.05% Tween-20 and 1% BSA in PBS). After incubation for 1 h at 37°C, plates were washed with wash buffer (0.05% Tween-20 in PBS). Next, HRP conjugated secondary antibodies for measurement of total IgG and individual IgG subclasses 1–4 (SouthernBiotech) were diluted according to the manufacturer’s instructions and incubated for 1 h. The plates were developed using TMB-core (AbD Serotec), and the OD_450 nm_ of each well was read using a CLARIOstar (BMG Labtech).

### Statistical analysis

Prism v 9.2.0 (GraphPad) was used for the analysis of ELISA data using an unpaired *t*-test of significance and analysis of Δ*rmpM* SBA data using a paired *t*-test of significance. For the comparison of clinical data between the participants with blocking sera and the total population, a two-proportion *z*-test with a 95% confidence interval was applied, with the resultant *z*-score used to calculate statistical significance using a two-tailed *z*-test. Results of statistical analysis throughout are represented as *P* ≤ 0.05 (*), *P* ≤ 0.01 (**) and *P* ≤ 0.001 (***).

## RESULTS

### Screening a collection of clinical *N. gonorrhoeae* isolates for resistance to autologous host serum

**Fig 1:**
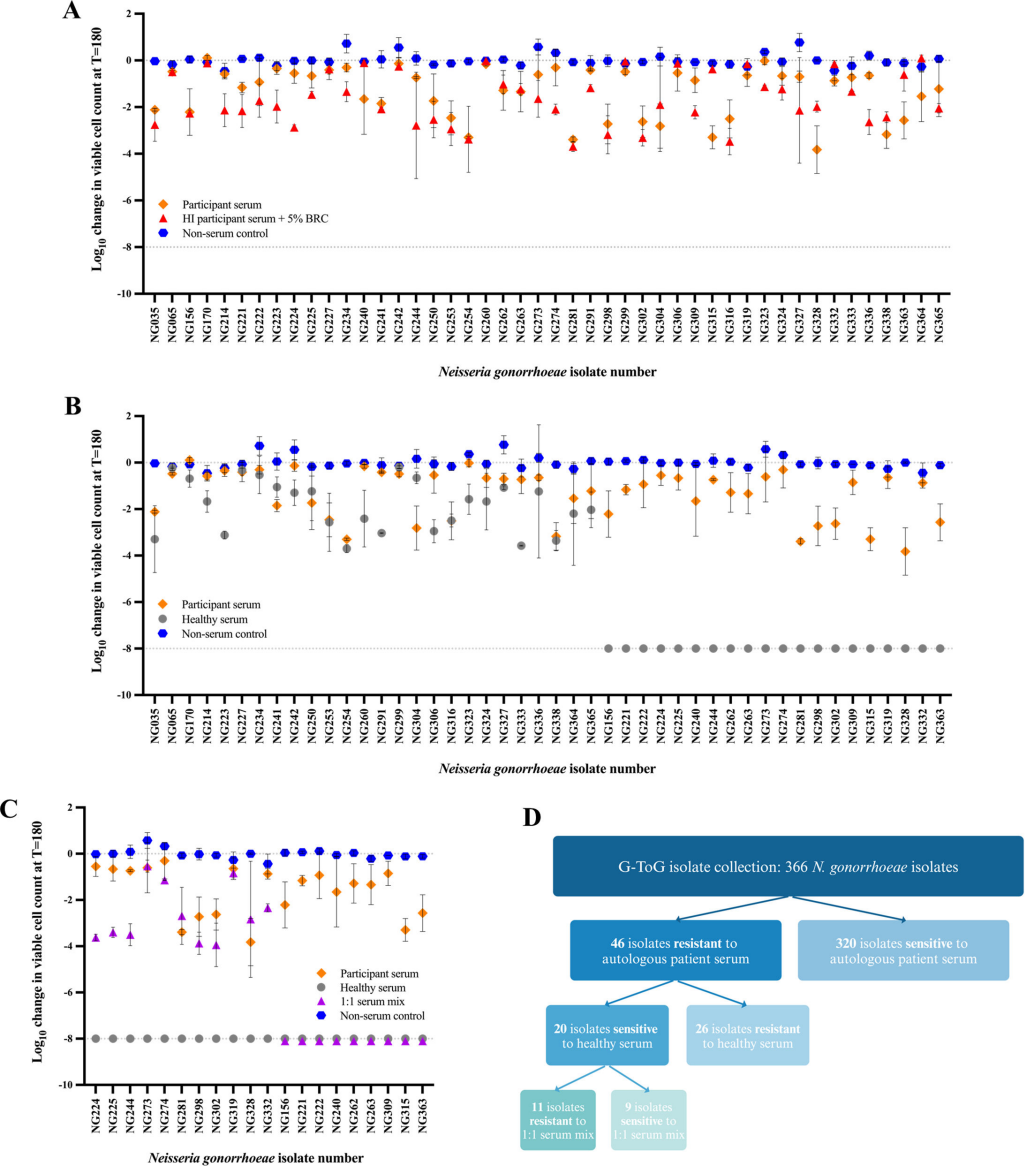
Screen of *N. gonorrhoeae* clinical isolates for their susceptibility to serum killing. A serum bactericidal assay (SBA) was used to screen 336 *N*. *gonorrhoeae* isolates collected during the G-ToG trial to determine their susceptibility to serum killing. Bacterial suspensions of each isolate were incubated with either the autologous participant serum, healthy control sera (HCS), a 1:1 mixture of both sera, HI participant sera + 5% BRC, or PBS as a negative control. The change in viable CFUs for isolates that were still viable after incubation for 180 min with indicated sera was measured, with negative values corresponding to a decrease in viable bacteria when compared to the initial inoculum, where a log_10_ kill of greater than 8 indicates the limit of detection for samples from which no colonies formed. (**A**) The survival of 46 *N*. *gonorrhoeae* clinical isolates in their autologous participant serum after 180 min incubation. (**B**) The same 46 *N*. *gonorrhoeae* clinical isolates that resisted killing by their autologous participant serum were incubated with HCS to determine survival. (**C**) The resultant 20 *N*. *gonorrhoeae* clinical isolates that were sensitive to killing by HCS were assayed to determine the effect of a 1:1 mix of both sera on survival. (**D**) Funnel diagram illustrates the screening process narrowing from 336 to 11 isolates that were resistant to a 1:1 mix of host serum and HCS. All data shown are the mean and standard deviation from three independent experiments.

To determine whether infection with *N. gonorrhoeae* induces blocking antibodies, we examined a collection of clinical isolates (*n* = 336) from 283 participants with acute gonococcal infection obtained as part of the G-ToG clinical trial (19). Each isolate was screened for resistance to CMK by incubating bacteria with serum from the host from which the strain had been isolated (i.e., autologous sera). As expected, 84% (282/336) of gonococcal isolates were killed by autologous host serum, demonstrated by a log_10_ kill of > 8 after 3 h incubation (Fig. 1A). Survival of the remaining 54 isolates in autologous serum could have resulted from the loss of complement during processing of samples. To rule this out and to focus on the influence of antibody responses, an exogenous complement source, BRC (5%), was added to HI autologous sera and the survival of the 54 isolates assessed. Results identified eight isolates that were killed by BRC and therefore only appeared resistant because the human complement component in the autologous serum had been insufficient to mediate bacterial lysis. These eight isolates were not studied further. Therefore, we identified 46 isolates that were resistant to CMK, indicating that the autologous serum might contain blocking antibodies (Fig. 1A).

### Effects of pooled healthy sera on isolates that resisted killing by autologous host serum

Some of these 46 isolates might have survived incubation with host serum because they are intrinsically resistant to CMK, independent of specific antibodies. Such isolates should also be resistant to killing by pooled HCS from uninfected individuals with no known history of gonococcal infection. SBAs were therefore repeated but using pooled HCS. The 46 isolates screened fell into two groups: 26 of the isolates resisted killing by HCS, which suggests that these isolates are intrinsically resistant to serum killing. The remaining 20 isolates were completely killed by HCS (Fig. 1B). As these 20 isolates are sensitive to HCS-mediated killing, but resistant to killing by autologous sera, the sera from these participants merited further investigation for the presence of blocking antibodies.

Blocking antibodies are typically defined by their ability to prevent HCS-mediated killing (8). Thus, each of the 20 isolates was incubated with a 1:1 mixture of HCS and the autologous serum (Fig. 1C). Nine of the 20 isolates were killed by the mixed sera, suggesting that the participants had failed to generate an effective immune response against the infecting isolate. However, 11 isolates either survived completely or significantly more in the presence of mixed sera than in HCS alone. The autologous sera therefore protected these 11 isolates from killing by HCS. Of note, some isolates were derived from the same participant but from different anatomical sites, and therefore these 11 isolates were isolated from 9 of the 283 participants recruited to the trial.

Previous studies have linked the development of blocking antibodies with certain clinical features associated with infection (9, 10, 12). Therefore, we examined whether patients with protective sera were associated with any of the clinical or demographic metadata. There were no significant differences in the proportion of participants with blocking/protective sera with ethnicity, gender, previous infection, HIV status, and symptomatic versus asymptomatic infection compared to the proportions within the whole cohort. However, there was a significant increase in the proportion of participants with protective sera in the men who have sex with women (MSW) sexual network (44% vs 21%) and with urethral infection (89% vs 55%) compared to the whole cohort ( Table S2).

### Effect of RmpM on the blocking of CMK

**Fig 2:**
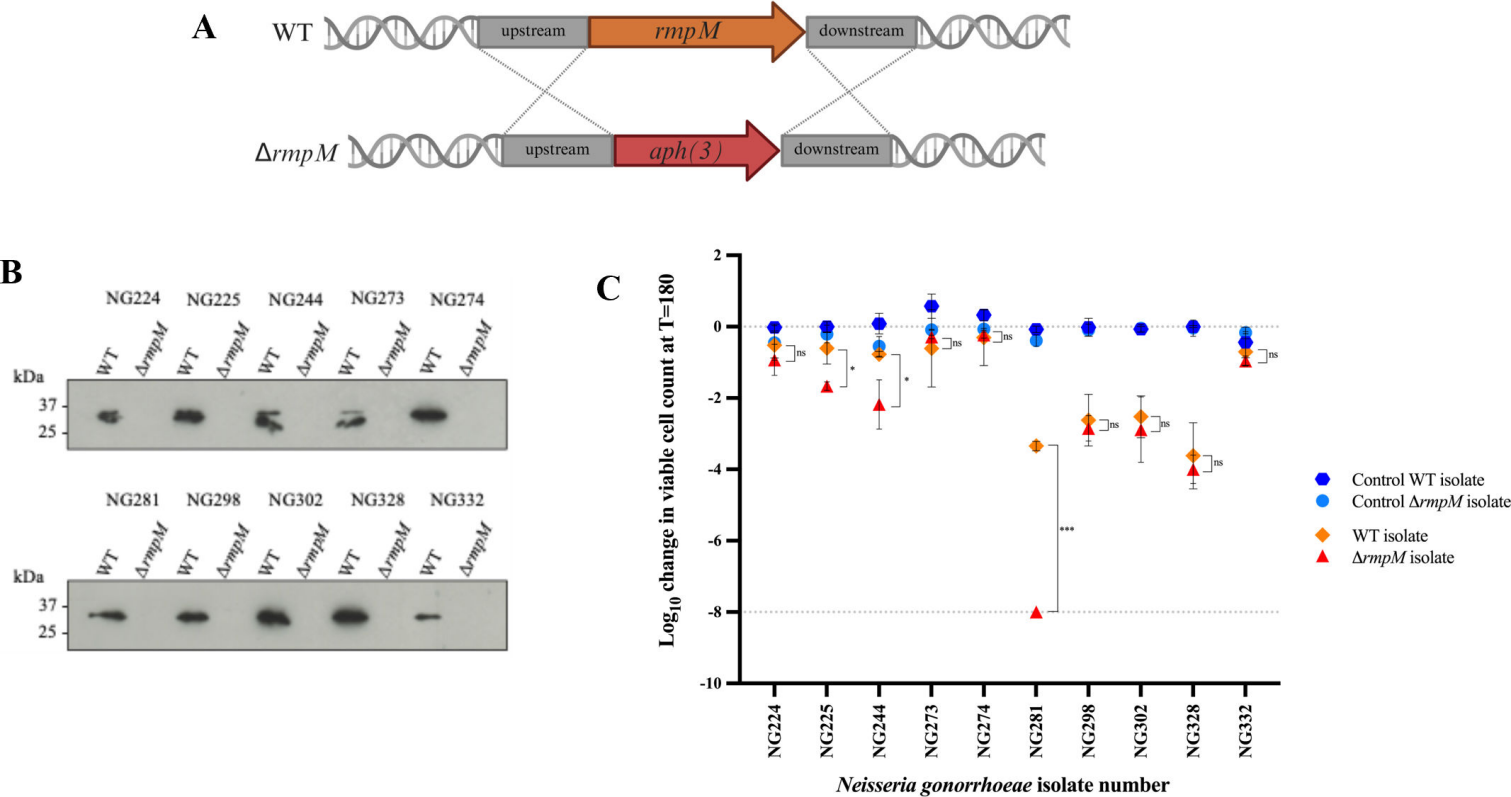
Involvement of RmpM in CMK. (**A**) Schematic representation of the deletion of *rmpM* by replacement with a kanamycin resistance cassette (*aph(3)*). Dotted lines represent the 1,000 bp regions upstream and downstream of *rmpM* for homologous recombination. (**B**) Western blot confirming deletion of *rmpM* using anti-RmpM antibodies. (**C**) A SBA was used to determine the survival of the WT and Δ*rmpM* mutant derivatives in autologous host serum. The change in viable CFUs for isolates that were still viable after incubation for 180 min with indicated sera was measured, with negative values corresponding to a decrease in viable bacteria when compared to the initial inoculum, where a log_10_ kill of greater than 8 indicates the limit of detection for samples from which no colonies formed. Statistical significance was measured using an paired *t*-test, where *, ***, and ns represent *P* ≤ 0.05, *P* ≤ 0.001, and non-significant, respectively.

Previous studies have identified RmpM as an antigen recognized by blocking antibodies. We therefore investigated whether blocking antibodies from patients with acute gonococcal infection within this study also targeted RmpM. To investigate this, insertional Δ*rmpM*::*aph(3)* mutants were generated in blocking isolates NG224, NG225, NG244, NG273, NG274, NG281, NG298, NG302, NG328, and NG332 and confirmed by PCR, sequencing and Western immunoblotting (Fig. 2B). A mutant for NG319 was not made due to a lack of autologous serum for future experiments. Each mutant was incubated with its orthologous serum to determine whether loss of RmpM restored sensitivity to CMK. Isolate NG281 Δ*rmpM*::*aph(3)* was completely killed by its orthologous serum after a 3 h incubation (Fig. 2C), while the isogenic mutants of the 9 remaining isolates were only slightly more sensitive to CMK than the corresponding parental strain (Fig. 2C). Therefore, we assume antibodies against RmpM play only a minor role in the blocking phenotype of these strains, with the mechanism remaining to be identified.

### Effect of antibody in blocking serum on CMK of *N. gonorrhoeae*

We next assessed whether serum IgG antibodies blocked CMK of the gonococcal isolates. To do this, total IgG antibodies were purified from sera of five participants from the nine identified (i.e., NG244-serum, NG298-serum, NG302-serum, NG273/274-serum, and NG332-serum). Total IgG was also purified from sensitive NG115-serum as a control. IgG from NG115-serum was tested in an SBA in the presence of 5% BRC, demonstrating that purified antibodies were still functional and able to initiate CMK (data not shown). Isolates were incubated with varying concentrations of autologous purified IgG mixed 1:1 with HCS; the highest antibody concentrations tested (1 mg/mL) were below normal physiological levels in the blood (6–16 mg/mL).

**Fig 3:**
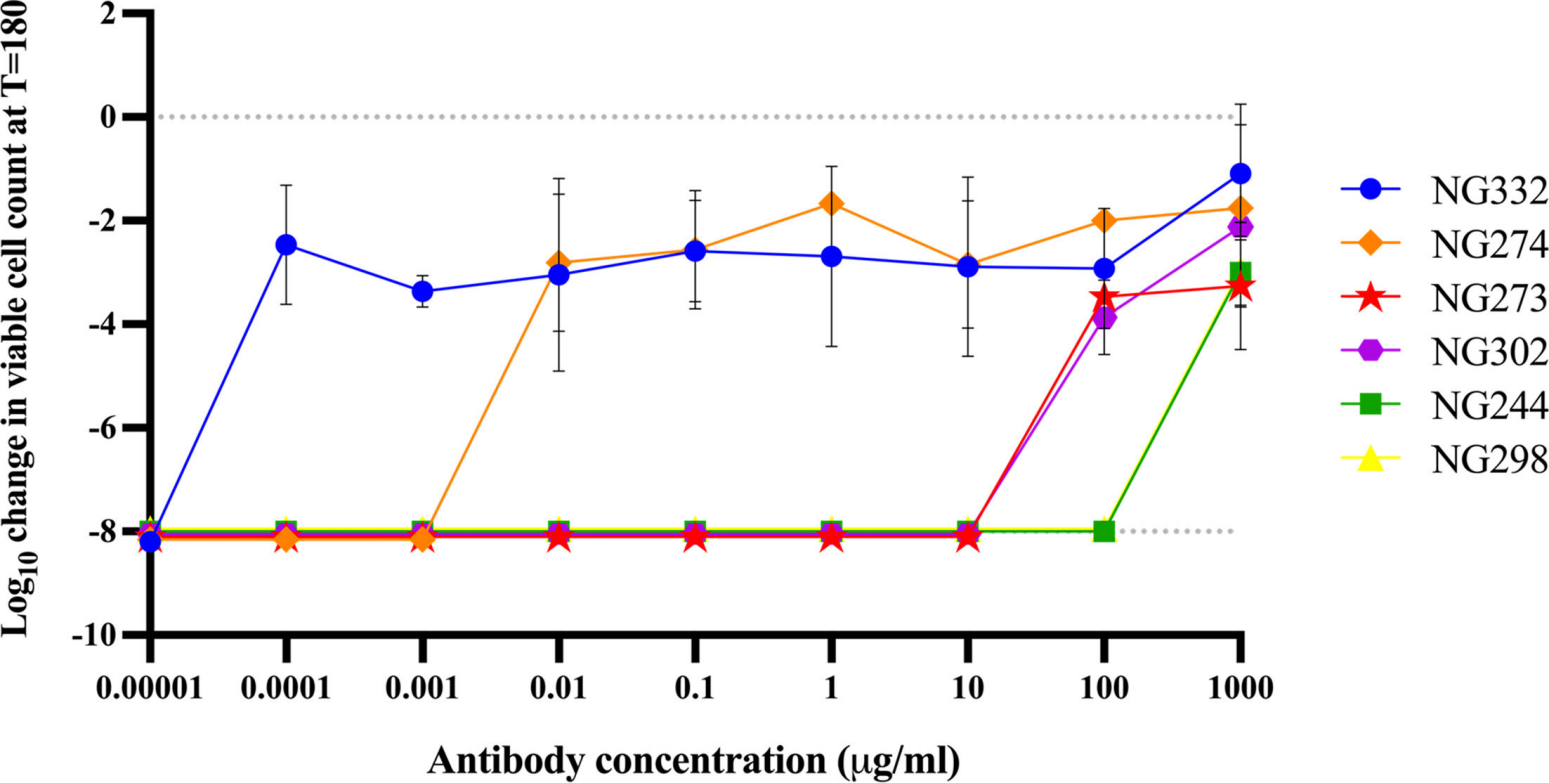
Effect of total IgG purified from blocking serum on autologous *N. gonorrhoeae* isolates. A SBA was used to determine the effect of total IgG antibodies purified from blocking serum on autologous clinical *N. gonorrhoeae* isolates. Bacterial suspensions of isolates NG332, NG274, NG273, NG302, NG244, and NG298 were incubated with a mixture of HCS and the indicated concentration of each autologous purified IgG antibody. The change in viable CFUs after 180 min was measured, with negative values corresponding to a decrease in viable bacteria when compared to the initial inoculum, where a log_10_ kill of greater than 8 indicates the limit of detection for samples from which no colonies formed. Data shown are the mean and standard deviation from two independent experiments.

As all isolates were sensitive to HCS-mediated killing, this modified SBA revealed whether total IgG could inhibit CMK. Of note, the highest concentrations of purified IgG (100–1,000 µg/mL) from autologous sera blocked isolates NG244, NG273, NG298, and NG302 from HCS-mediated killing (Fig. 3). Interestingly, significantly lower concentrations (from 0.0001 µg/mL) of IgG from NG274-serum and NG332-serum were able to block HCS-mediated killing of the corresponding isolates. This suggests that there might be isolate-related or IgG subclass-related differences driving this stronger phenotype.

### Effect of different IgG subclasses on blocking CMK

**Fig 4:**
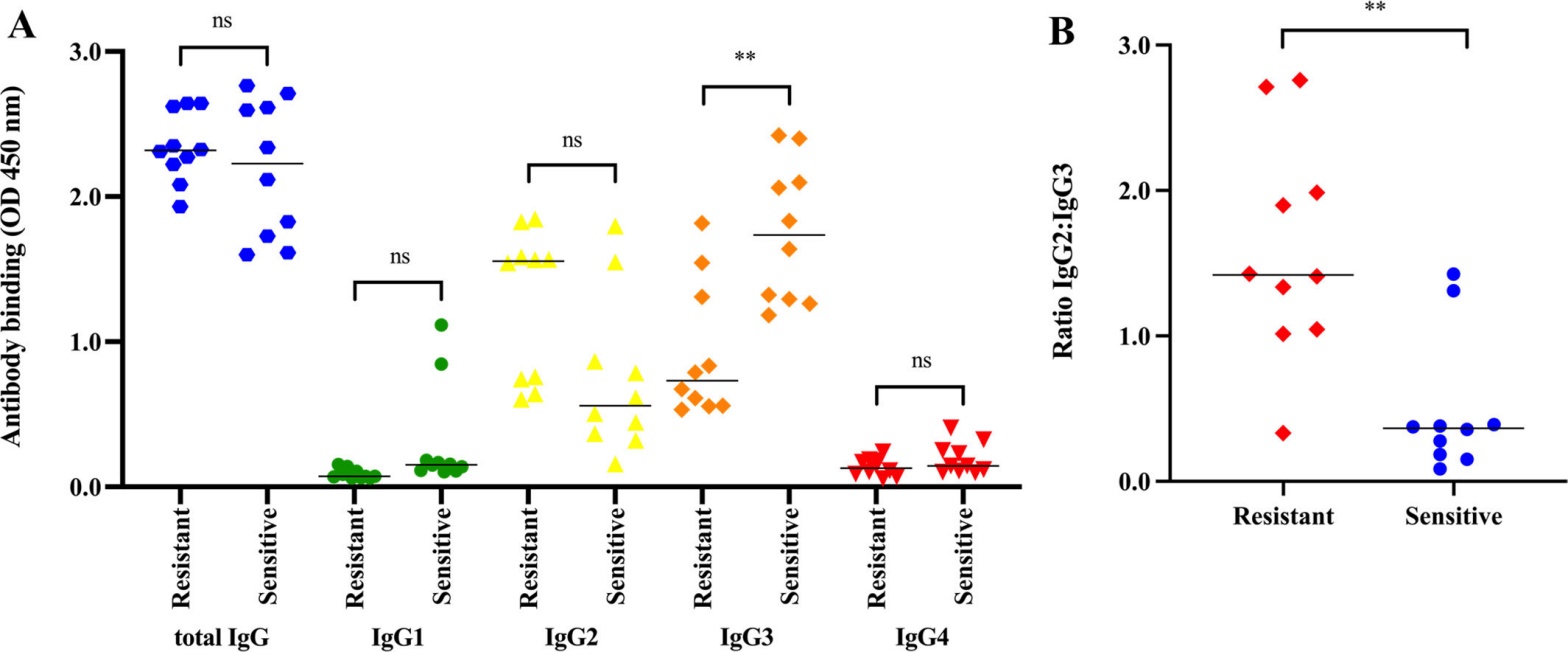
Comparison of IgG subclasses binding to serum-resistant and serum-sensitive isolates. An ELISA was used to investigate the amount of different IgG subclasses that bound to whole-cell *N. gonorrhoeae*. (**A**) Total IgG, IgG1, IgG2, IgG3, and IgG4 that bound to 10 resistant isolates from autologous blocking serum were measured and compared to the binding of the same subclasses to 10 isolates sensitive to autologous serum. (**B**) The IgG2:IgG3 ratio that bound each of the 10 resistant and sensitive isolates was calculated and compared. Data shown are the mean and standard deviation from two independent experiments. Statistical significance was measured using an unpaired *t*-test, where ** and ns represent *P* ≤ 0.01 and non-significant, respectively.

Given that the concentration of IgG which blocked HCS-mediated killing varied between isolates, we hypothesized that differences in the antigenic profile of isolates could alter the binding of IgG subclasses and subsequent serum resistance. To test this, whole-cell ELISAs were performed to determine binding of IgG subclasses to 10 gonococcal isolates resistant to the 1:1 serum mix (Fig. 1C). As controls, the same number of isolates that were sensitive to CMK were selected based on demographics matched with the participants with resistant isolates. There were no significant differences in binding of total IgG, IgG1, and IgG4 between the resistant and sensitive isolates (Fig. 4A). However, there was a trend of increased IgG2 binding to resistant isolates, although this did not reach statistical significance. Of note, there was a statistically significant decrease in binding of IgG3 to resistant isolates compared to sensitive isolates (Fig. 4A). Moreover, the IgG2:IgG3 ratio bound to resistant isolates was significantly increased compared with sensitive isolates (Fig. 4B), suggesting that the proportion of binding of these antibody subclasses to bacteria might determine serum susceptibility.

### Effect of blocking serum on the survival of serum-sensitive isolates

**Fig 5:**
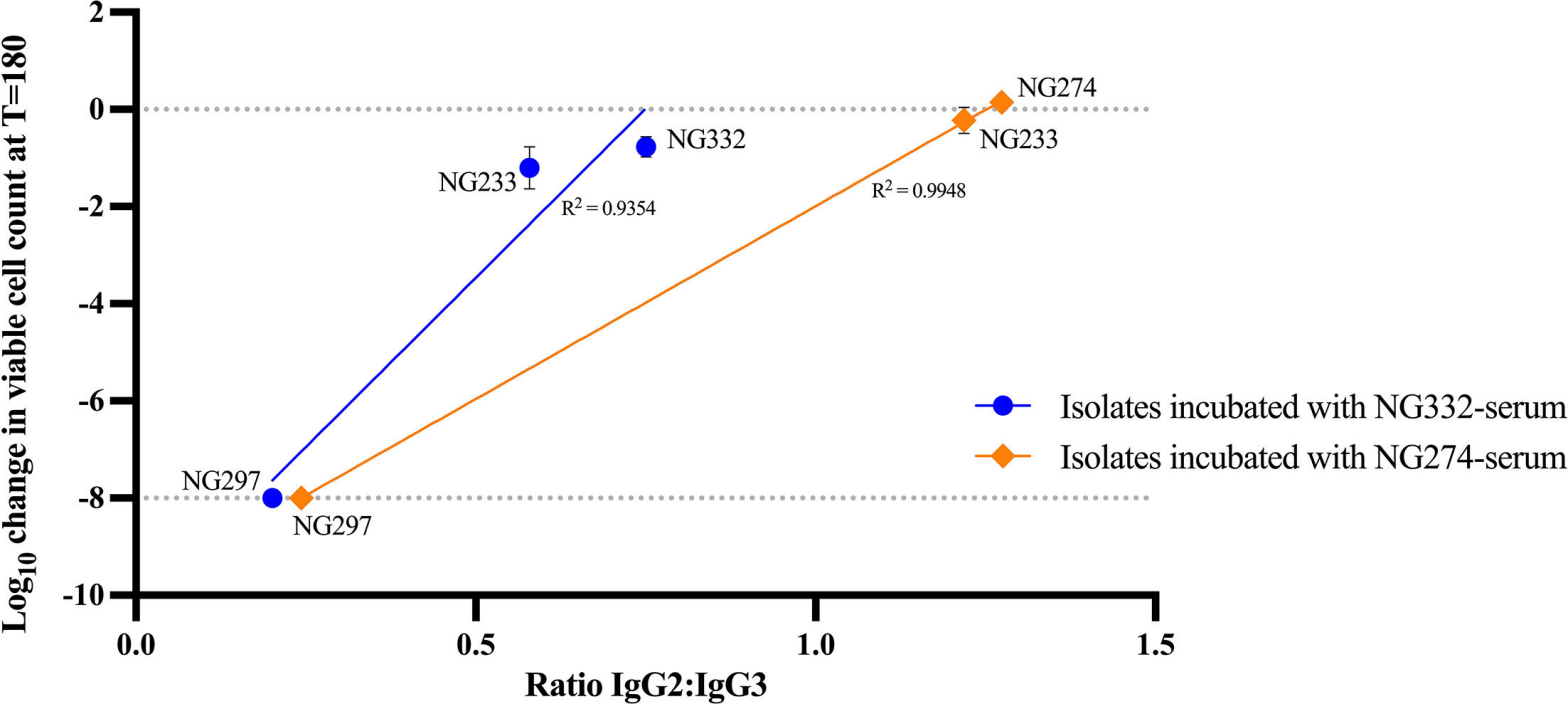
Effect of IgG2:IgG3 ratio binding to isolates on *N. gonorrhoeae* survival. Correlation of bacterial survival with the IgG2:IgG3 ratio bound to isolates NG332, NG274, NG233, and NG297 when incubated with blocking NG332-serum or NG274-serum. The change in viable CFUs after 180 min was measured, with negative values corresponding to a decrease in viable bacteria when compared to the initial inoculum. Values at −8 indicate the limit of detection for samples from which no colony formed even from the least diluted sample plated. Data shown are the mean and standard deviation from two independent experiments.

To determine whether blocking sera that contained a high ratio of IgG2:IgG3 antibody could block CMK of otherwise sensitive isolates, we incubated blocking sera with non-autologous sensitive isolates and quantified the level of IgG2:IgG3 binding to the cell surface of these isolates. To do this, we chose the blocking NG332-serum and NG274-serum and the sensitive isolates NG233 and NG297. Interestingly, both NG332-serum and NG274-serum protected NG233 isolate from CMK, but not NG297 (Fig. 5). Whole-cell ELISAs revealed that a higher IgG2:IgG3 ratio from blocking sera (NG332-serum and NG274-serum) was bound to isolate NG233 than NG297 (Fig. 5). These results provide further evidence that increased binding of IgG2 to the gonococcal cell surface can block bactericidal IgG3 from initiating CMK.

## DISCUSSION

Our investigation of the sensitivity of *N. gonorrhoeae* isolates to killing by autologous sera revealed that 3% (9/283) of participants recruited to the G-ToG trial produced blocking antibodies. In contrast, other studies (10, 13) reported that 1 in 3 adults with cystic fibrosis and 20% of patients with non-cystic fibrosis bronchiectasis had blocking antibodies against *P. aeruginosa*, respectively. In addition, 24% of patients with *E. coli* urosepsis had blocking antibodies (12). These data suggest that the prevalence of blocking antibodies during chronic infection is relatively high. In this study, however, all gonococcal infections were acute, and the incidence of blocking antibodies was relatively low. The driver for infection not generating bactericidal sera could be at the transcriptional level in these isolates and/or mediated by fundamental differences in the host response to infection. Interestingly, we observed that participants with urethral infection and those within the MSW sexual network were found to be significantly more likely to have protective sera. Thus, it will be interesting to determine the clinical implications of blocking antibodies on recurrent gonorrhea and outcomes in different patient groups in follow-up studies.

We found that IgG can block CMK in a concentration-dependent manner. These data add to the growing body of evidence implicating IgG antibodies in blocking CMK following Gram-negative bacterial infection in some individuals (9–13, 22–24). In general, the mechanism by which antibodies block CMK has not yet been characterized, although multiple theories have been proposed, as further detailed in a recent review (8). We show that the resistance of some gonococcal isolates to killing by autologous serum is related to an imbalance of the ratio of IgG2:IgG3 binding to the bacterial cell surface. This is distinct from other Gram-negative bacteria, where only very high titers of IgG2 binding to bacteria correlated with the blocking phenotype (9, 10, 12). Blocking antibodies against *N. gonorrhoeae* have been observed against the outer membrane protein RmpM in patients infected with DGI strains (18). DGI is caused by a distinct subset of gonococcal strains (25), and our data presented here suggest that RmpM does not block CMK from patients with acute gonococcal infection used in this study (Fig. 2). Thus, it is likely that the IgG2 identified in this study targets an alternative antigen, given the preference of IgG2 for binding to polysaccharide antigens (26), such as capsules or lipooligosaccharide. Although outside the scope of this study, further work will characterize the antigens targeted by both the IgG2 and IgG3 antibodies identified here.

The cross-protection observed against *N. gonorrhoeae* by the OMV vaccine, MeNZB (7), has revitalized efforts to develop vaccines against gonorrhea. Therefore, the importance of assessing the IgG2:IgG3 antibody ratios bound to the bacterial cell surface should be considered. In a vaccine, it would be preferable to include *N. gonorrhoeae* antigens (even alone or in OMVs) that elicit bactericidal IgG3 antibodies, compared to antigens that stimulate blocking IgG2 responses. This should generate a more efficient antibody-driven CMK, while limiting the production of blocking antibodies in vaccinated individuals. Many studies have suggested that a vaccine evoking a Th1 response is important for immune memory and clearance of gonococcal infection (27–29), unlike the Th17 response produced during natural infection (30). Th1 development causes plasma cells to secrete high-affinity specific IgG3 antibodies (31). As increased IgG3 binding correlated with bacterial killing, our results further emphasize that IgG3 responses might be important for protection against gonococcal infection and offer a correlate of immunity. Thus, inclusion of antigens that stimulate this response may be essential for vaccine-induced protection.

In summary, we provide evidence for a new mechanism to explain why a minority of participant antisera protect some gonococcal strains from CMK and, conversely, why the majority kill this widespread pathogen. Our data reveal that these blocking antibodies could be predictive of clinical outcome and/or repeat infections but also provide fundamental information that is informative for the development of effective vaccines against gonorrhea.

## SUPPLEMENTAL MATERIAL

Tables S1 and S2 mbio.03367-23-s0001.docx Primer information and patient demographics.

ASM does not own the copyrights to Supplemental Material that may be linked to, or accessed through, an article. The authors have granted ASM a non-exclusive, world-wide license to publish the Supplemental Material files. Please contact the corresponding author directly for reuse.
