# Supplementary material for: Inhibition of Neisseria gonorrhoeae complement-mediated killing during acute gonorrhea is dependent upon the IgG2:IgG3 antibody ratio
Source: mBio. 2026 Jan 30;17(3):e03367-23. doi: 10.1128/mbio.03367-23 (PMC12977606; doi:10.1128/mbio.03367-23)
Supplement: Tables S1 and S2 — Primer information and patient demographics. [file mbio.03367-23-s0001.docx]

**Supplementary Table 1**. **Primers used in this study**

**Supplementary Table 2**. **Comparisons of clinical metadata between the whole population and participants with blocking sera.** Proportions of participants recruited to the G-ToG clinical trial in the indicated categories amongst the whole population (n=283) versus the participants with blocking sera (n=9). Statistical significance was calculated using a 2-tailed z-test. Significant values (*p*<0.05) are shown in red bold text.
